# Chemotherapy-induced peripheral neuropathy in children and adolescent cancer patients

**DOI:** 10.3389/fmolb.2022.1015746

**Published:** 2022-10-14

**Authors:** Nicolette Tay, E-Liisa Laakso, Daniel Schweitzer, Raelene Endersby, Irina Vetter, Hana Starobova

**Affiliations:** ^1^ Institute for Molecular Bioscience, The University of Queensland, St Lucia, QLD, Australia; ^2^ Mater Research Institute-The University of Queensland, South Brisbane, QLD, Australia; ^3^ Telethon Kids Institute, University of Western Australia, Nedlands, WA, Australia; ^4^ The School of Pharmacy, The University of Queensland, Woolloongabba, QLD, Australia

**Keywords:** chemotherapy-induced peripheral neuropathy, pediatric cancer patients and survivors, neuroinflammation, pharmacological and non-pharmacological treatment strategies, CIPN mechanisms

## Abstract

Brain cancer and leukemia are the most common cancers diagnosed in the pediatric population and are often treated with lifesaving chemotherapy. However, chemotherapy causes severe adverse effects and chemotherapy-induced peripheral neuropathy (CIPN) is a major dose-limiting and debilitating side effect. CIPN can greatly impair quality of life and increases morbidity of pediatric patients with cancer, with the accompanying symptoms frequently remaining underdiagnosed. Little is known about the incidence of CIPN, its impact on the pediatric population, and the underlying pathophysiological mechanisms, as most existing information stems from studies in animal models or adult cancer patients. Herein, we aim to provide an understanding of CIPN in the pediatric population and focus on the 6 main substance groups that frequently cause CIPN, namely the vinca alkaloids (vincristine), platinum-based antineoplastics (cisplatin, carboplatin and oxaliplatin), taxanes (paclitaxel and docetaxel), epothilones (ixabepilone), proteasome inhibitors (bortezomib) and immunomodulatory drugs (thalidomide). We discuss the clinical manifestations, assessments and diagnostic tools, as well as risk factors, pathophysiological processes and current pharmacological and non-pharmacological approaches for the prevention and treatment of CIPN.

## 1 Introduction

Chemotherapy-induced peripheral neuropathy (CIPN) is defined as dysfunction of peripheral nerves caused by the use of one or more chemotherapeutic agents. The prevalence of CIPN amongst pediatric patients and cancer survivors can approach >90% depending on the patient population, chemotherapeutic agent used, the cumulative dose administered, and the criteria used to diagnose CIPN (Purser et al., 2014; Kandula et al., 2016). While all main classes of chemotherapy agents are known to cause CIPN, vinca alkaloids and platinum compounds are used more commonly in pediatric populations and are highly associated with pediatric CIPN (Kandula et al., 2016). These agents affect the function of the sensory, motor and/or autonomic peripheral nervous systems, leading to the development of debilitating and untreatable symptoms. While CIPN caused by the different classes of chemotherapeutics can manifest with similar symptoms, there are key differences in the underlying molecular mechanisms of neurotoxicity (Starobova and Vetter, 2017). These symptoms are debilitating as they can persist for months or years after the discontinuation of the chemotherapy treatment, resulting in a need to develop effective treatment strategies for CIPN to improve the long-term functional performance and quality of life of pediatric patients with cancer. However, we lack a detailed understanding of the underlying mechanism of CIPN in children that is essential for the development of effective prevention or treatment strategies (Kandula et al., 2016; Kandula et al., 2018). In the treatment of childhood cancer, chemotherapy is often administered as a complex regimen that combines multiple drugs, causing synergistic neurotoxicity and complicating the identification of specific therapeutic targets. Despite past efforts to develop effective treatments for CIPN, no successful approach has been identified to date. Instead, the amelioration of symptoms in pediatric patients often relies on the reduction, interruption, or cessation of the chemotherapy treatment, drastically decreasing their likelihood of survival (Grisold et al., 2012; Smith et al., 2021).

This review focusses on the 6 main substance classes of chemotherapeutics that frequently cause CIPN, the vinca alkaloids (vincristine), platinum-based antineoplastics (cisplatin, carboplatin and oxaliplatin), taxanes (paclitaxel and docetaxel), epothilones (ixabepilone), proteasome inhibitors (bortezomib), and immunomodulatory drugs (thalidomide). While there is data to suggest that the drugs doxorubicin and cyclophosphamide, often used in the treatment of childhood leukemia or brain cancers, may cause neuropathy, there is insufficient evidence of this in pediatric populations and therefore will not be included in this review (Lipshultz et al., 1991; Mulrooney et al., 2009). We discuss the clinical manifestations, risk factors and pathophysiological processes involved in pediatric CIPN. Specifically, we highlight the unique features of pediatric CIPN compared to adult CIPN and discuss diagnostic tools for childhood CIPN. Finally, we critically evaluate the efficacy of current pharmacological and non-pharmacological approaches for the prevention and treatment of pediatric CIPN and discuss future advances in development of CIPN treatments.

## 2 Childhood and adolescent cancers

The most common types of cancer diagnosed in children <14 years include leukemias and cancers of the central nervous system (CNS) (for full list see Table 1) (Siegel et al., 2022). On the other hand, the most common types of cancer diagnosed in adolescents aged 15 to 19 are Hodgkin’s lymphoma and thyroid cancer (for full list see Table 1) (Siegel et al., 2022). It has been predicted that by 2030, 6.7 million children will be diagnosed with cancer worldwide (Ward et al., 2019). Despite the significant improvement in the 5-year relative survival, from 73% in 1994 to 86% in 2016, cancer remains the leading cause of death by disease for children and adolescents in first-world countries (Youlden et al., 2015). Unlike adult cancers, which are predominantly caused by combination of environmental (physical, chemical and biological) carcinogens with genetic factors, there is no evidence that environmental factors cause childhood cancer (Soto and Sonnenschein, 2010; de Martel et al., 2020). Existing data suggest that the presence of certain genetic risk factors may be the cause, however, further studies in pediatric populations are required (Daub et al., 2021). The type of cancer, the extent of spread, the response of the tumour to chemotherapy treatment and the activity of the immune system distinguish pediatric cancers from adult tumors, necessitating that these malignancies are approached as separate diseases (Ries, 1999). The standard of care therapeutics for cancers such as leukemia and brain cancer include neurotoxic chemotherapeutics that cause dose-limiting, long-term adverse effects, decreasing the overall survival (Smith et al., 2021).

**TABLE 1:** Common types of cancer arising in children and adolescents (Siegel et al., 2022).

Types of cancer in children 0–14 years	Percentage of cases (%)	Types of cancer in adolescents 15–19 years	Percentage of cases (%)
Leukemia (acute lymphoblastic leukemia and acute myeloid leukemia)	29	Hodgkin’s lymphoma	15
Central nervous system tumors	26	Thyroid cancer	11
Neuroblastoma	6	Central nervous system tumors	10
Wilm’s tumor	5	Acute lymphoblastic leukemia	8
Non-Hodgkin’s lymphoma	5	Non-Hodgkin’s lymphoma	8
Hodgkin’s lymphoma	3	Testicular cancer	8
Rhabdomyosarcoma	3	Soft tissue sarcoma	7
Retinoblastoma	2	Bone tumors (osteosarcoma and Ewing’s sarcoma)	7
Osteosarcoma	2	Melanoma	6
Ewing’s sarcoma	1	Acute myeloid leukemia	4

## 3 The use and mode of action of chemotherapy drugs

The six following chemotherapeutic classes are indicated for the treatment of pediatric cancers (for full list see Table 2). Vinca alkaloids are a class of drugs derived from the Madagascar periwinkle plant, *Catharanthus roseus G. Don*. The 4 major vinca alkaloids that are used clinically are vincristine (first generation), vinblastine (first generation), vindesine (second generation) and vinorelbine (third generation). The third-generation agents are reportedly less neurotoxic and associated with a decreased frequency of adverse events (Budman, 1997). Vinca alkaloids are mitotic inhibitors, and are frequently used in pediatric chemotherapy regimens, particularly for blood cancers and solid tumors. Vinca alkaloids cause apoptosis of cancer cells by binding to the β-tubulin subunit of microtubules and to the spindle proteins in the S phase of the cell cycle, inhibiting microtubule formation, which results in mitotic arrest of the cancer cells in metaphase (Himes, 1991; Downing, 2000).

**TABLE 2:** Summary of the mode of action and incidence of pediatric CIPN associated with various chemotherapeutic agents. WHO scale of CIPN grades: Grade 1: paresthesia and/or decreased tendon reflexes; Grade 2: severe paresthesia and/or mild weakness, Grade 3: intolerable paresthesia and/or marked motor loss; Grade 4: paralysis (Miller et al., 1981).

Cancer type		Mode of action on cancer cells	Incidence	References
Vincristine	• Acute lymphoblastic leukemia	Binds to tubulin and inhibits microtubules formation resulting in mitotic arrest at metaphase	Any grade: 78%–100%	Anghelescu et al. (2011), Langholz et al. (2011), Lavoie Smith et al. (2015)
	• Hodgkin’s lymphoma			
	• Non-Hodgkin’s lymphoma		Grade 3/4: 10%–52%	
	• Neuroblastoma			
	• Rhabdomyosarcoma			
	• Wilms’ tumor			
	• Embryonal tumors of the CNS (medulloblastoma, atypical teratoid/rhabdoid tumor (AT/RT), pineoblastoma, etc)			

Oxaliplatin	• Refractory or relapsed solid tumors	Binds to DNA, forming cross-links that prevent DNA replication, transcription, leading to cell cycle arrest	Grade 1/2: 37%–50%	Fouladi et al. (2006), Spunt et al. (2007a), Macy et al. (2013)
			Grade 3/4: 3%–8%	
Cisplatin	• Relapsed and refractory lymphoma	Binds to DNA, forming cross-links that prevent DNA replication, transcription, leading to cell cycle arrest	Unclear as existing evidence is mostly from case reports	Kandula et al. (2016)
	• Low-grade gliomas			
	• Embryonal tumors of the CNS (medulloblastoma, AT/RT, pineoblastoma)			
	• Neuroblastoma			
	• Retinoblastoma (Frequently used in combination with vincristine)			
Carboplatin	• Solid tumors	Binds to DNA, forming cross-links that prevent DNA replication, transcription, leading to cell cycle arrest	Grade 1/2: 4%	Loss et al. (2004)
	• Low-grade glioma			
	• Retinoblastoma			
Paclitaxel	• Under evaluation for recurrent or refractory solid pediatric tumors	Microtubule stabilizing agent, causes G2/M cell cycle arrest	Grade 1/2: 11%–50%	Hurwitz et al. (1993), Doz et al. (2001), Horton et al. (2008), Geller et al. (2009)
			Grade 3/4: 6%–12%	
Docetaxel	• Under evaluation for recurrent or refractory solid pediatric tumors	Inhibits microtubules disassembly, resulting in G2/M cell cycle arrest and cell death	Grade 3/4: 5%	Blaney et al. (1997), Seibel et al. (1999), Zwerdling et al. (2006), Yoon et al. (2014)
Ixabepilone	• Under evaluation for refractory solid pediatric tumors	Suppresses microtubules dynamics, resulting in G2/M cell cycle arrest and cell death	Grade 1/2: 22%	Widemann et al. (2009), Jacobs et al. (2010)
			Grade 3/4: 2%–5%	
Bortezomib	• Relapsed leukemias and refractory lymphomas	Reversibly inhibits the 26S proteasome resulting in disruption of various cell signaling pathways and cell cycle arrest	Grade 1/2: 10%–18%	Blaney et al. (2004), Muscal et al. (2013), Horton et al. (2014)
			Grade 3/4: 6%	
Thalidomide	• Medulloblastoma	Inhibitor of angiogenesis, prevents the production of interleukin-6, activates apoptotic pathways via caspase 8-mediated cell death	Grade 2 or greater: 20%–40%	Priolo et al. (2008), Lazzerini et al. (2013), Lazzerini et al. (2015)
	• Hepatocellular carcinoma			

Taxanes inhibit microtubule polymerization, leading to inhibition of their function and mitosis, followed by apoptosis of cancer cells (Schiff and Horwitz, 1980; Downing, 2000). Paclitaxel and docetaxel are only recommended to be used for the treatment of refractory or recurrent solid pediatric tumors (Hurwitz et al., 1993). However, phase I and II studies in pediatric populations show that docetaxel elicits only partial response in Ewing sarcoma and shows low efficacy in other solid tumor types, such as medulloblastoma and neuroblastoma (Oppert et al., 1991; Seibel et al., 1999).

**FIGURE 1:**
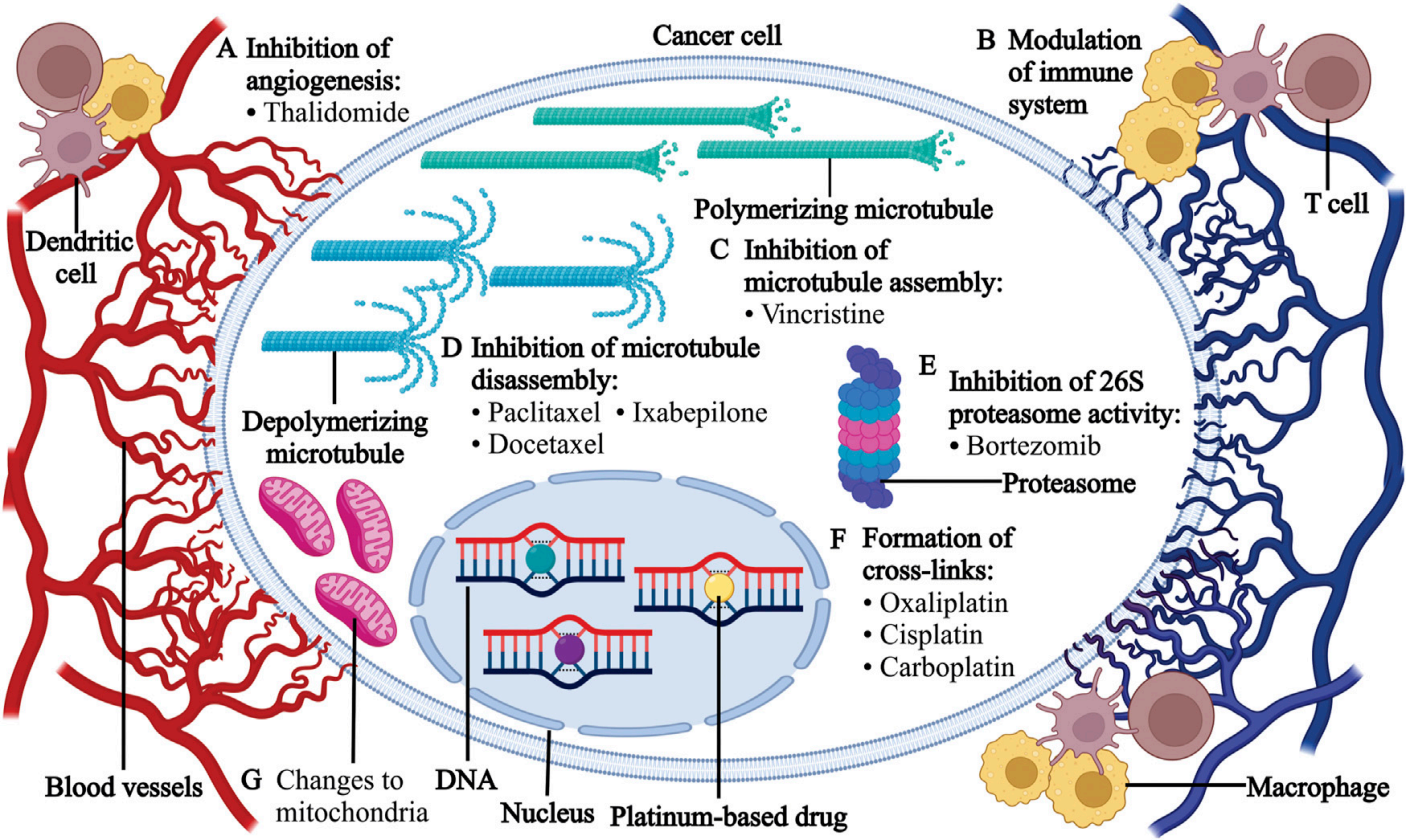
Mode of action of chemotherapeutic agents in cancer cells. **(A)** Thalidomide inhibits angiogenesis, prevents the production of interleukin-6 and activates apoptotic pathways *via* caspase 8-mediated cell death. **(B)** Chemotherapy affects the tumor immune microenvironment. **(C)** Vincristine binds to the β-tubulin subunit of microtubule in the S phase of the cell cycle, which leads to the inhibition of microtubule assembly. The disruption of mitotic spindle formation results in the mitotic arrest of cancer cells at metaphase and subsequent cell death. **(D)** Paclitaxel, docetaxel and ixabepilone bind to the β-tubulin subunit of microtubule and inhibit microtubule disassembly, which causes G2/M cell cycle arrest and cell death. **(E)** Bortezomib reversibly inhibits the 26S proteasome, which disrupts proteasome-mediated proteolysis. This disruption causes the accumulation of ubiquitinated proteins and subsequent cell death. **(F)** Oxaliplatin, cisplatin and carboplatin bind to DNA to form cross-links that prevent DNA replication and transcription, leading to cell cycle arrest and apoptosis. **(G)** Vincristine, paclitaxel and docetaxel alter the mitochondrial electron transport chain while cisplatin results in the increased production of reactive oxygen species.

Epothilones are another group of microtubule-stabilizing agents that cause mitotic arrest. Ixabepilone, a semi-synthetic analog of epothilone B, is used in the treatment of refractory breast cancers in adults and is being evaluated for use with pediatric refractory solid tumors given encouraging preclinical data (Low et al., 2005; Jacobs et al., 2010; Genovesi et al., 2021) (Figure 1).

Platinum-based compounds, particularly cisplatin, carboplatin and oxaliplatin, cause apoptotic cell death by binding to DNA strands leading to cancer cell arrest and cell death. They are frequently used in the treatment of solid tumors in the adult and pediatric population (Szymkowski et al., 1992; Mello et al., 1995).

Bortezomib is a dipeptide derivative of boronic acid that inhibits the mammalian 26S proteasome and the secretion of cytokines in the bone marrow (Kane et al., 2003). Consequently, it induces apoptosis and interferes with downstream signaling pathways such as angiogenesis and cell proliferation (Hideshima et al., 2001). It is the first proteasome inhibitor that was approved by the Food and Drug Administration (FDA) for the treatment of multiple myeloma and mantle cell lymphoma and is used to treat relapsed leukemias and refractory lymphomas in the pediatric population (Bertaina et al., 2017).

Thalidomide, an immunomodulatory drug, is a potent vascular endothelial growth factor (VEGF) inhibitor, and it is frequently used to treat multiple myeloma in adults (Franks et al., 2004; Kenyon et al., 1997; D'Amato et al., 1994). It is used to treat inflammatory conditions such as juvenile rheumatoid arthritis, vasculitis, refractory inflammatory bowel disease, graft versus host disease, and less commonly, medulloblastoma and hepatocellular carcinoma in pediatric patients (Kandula et al., 2016).

## 4 Incidence of pediatric chemotherapy-induced peripheral neuropathy

Pediatric CIPN is a common, debilitating side effect of chemotherapy and the actual incidence in pediatric populations is probably higher than the reported incidence (Table 2). Especially in very young pediatric populations, CIPN is often un- or under-recognized, as young children are less able to verbally communicate and, as such, the diagnosis often relies on reporting performed by a parent/care-giver, or using unstandardized diagnostic tools and assessments of CIPN (Calhoun et al., 2003; Gilchrist et al., 2014). Additionally, the extent of neuronal damage and the severity of symptoms depends on the chemotherapeutic agents used, the cumulative dose of the chemotherapy, the chemotherapy regimen, the duration of the chemotherapy, the site of the neuronal damage (e.g., myelin sheath, axon, cell body) and concomitant neuropathies and diseases (Kandula et al., 2016).

The incidence of vincristine-induced peripheral neuropathy in children highly depends on the cumulative dose of vincristine and was reported as high as 90% (Anghelescu et al., 2011; Langholz et al., 2011; Lavoie Smith et al., 2015; Kandula et al., 2016). For example, only 10% of patients who received cumulative doses of 5–10 mg/m^2^ of vincristine experienced severe peripheral neurotoxicity compared to 20%–52% of patients who received cumulative doses 30 mg/m^2^ (Langholz et al., 2011). Additionally, conventional nerve conduction studies established that >90% of patients who were given vincristine had motor neuropathy after 4–5 weeks of treatment (Courtemanche et al., 2015a). The differences in reported incidence from these studies emphasize that the diagnosis of CIPN often depends on the diagnostic tools used, and that CIPN is likely widely under-reported.

Oxaliplatin is the most neurotoxic compound in the platinum-based group, with incidence of CIPN reported to be as high as 50% (Fouladi et al., 2006; Spunt et al., 2007a; Macy et al., 2013). Cisplatin and carboplatin are less neurotoxic compared to oxaliplatin, however, the incidence of cisplatin or carboplatin-induced neuropathy in the pediatric population is rather unclear, as there is lack of clinical studies and both derivates are almost exclusively administered with other chemotherapy agents, such as vincristine or paclitaxel (Kandula et al., 2016).

Paclitaxel was reported to cause CIPN in up to 50% of pediatric patients, however the cumulative dose and neurotoxicity relationship is unclear (Hurwitz et al., 1993; Doz et al., 2001; Horton et al., 2008; Geller et al., 2009). On the other hand, only 5% of pediatric patients receiving docetaxel developed CIPN, particularly those that were administered 4 or more courses of docetaxel (Blaney et al., 1997; Seibel et al., 1999; Zwerdling et al., 2006; Yoon et al., 2014).

Phase I and II studies investigating the use of bortezomib in pediatric patients have shown that 10%–18% of children who were given 1–2 courses of bortezomib experienced grade 1 or 2 CIPN and up to 6% of children experienced Grade 3 or 4 CIPN (Blaney et al., 2004; Muscal et al., 2013; Horton et al., 2014).

While there is a lack of toxicity data in children for epothilones, data from adult clinical trials has shown that up to 67% of patients that received ixabepilone experienced sensory, motor and autonomic neuropathy (Vahdat et al., 2012). It remains a challenge to estimate the incidence of neurotoxicity caused solely by ixabepilone as patients who are treated with this agent have typically been given other neurotoxic treatments. Due to the lack of evidence and information on long-term effects, the exact activity of ixabepilone in the peripheral nervous system and reversibility of neuropathy after the cessation of treatment remain unknown. However, the few studies involving children that have been reported suggest that ixabepilone causes grade 1 sensory neuropathy in up 22% of cases, although the number of patients examined was limited (Widemann et al., 2009; Jacobs et al., 2010).

Similarly, the data assessing thalidomide-induced neuropathy in the pediatric population is scarce. Two clinical studies have shown that 20%–40% of patients receiving a dose of 1.5–3 mg/kg/day of thalidomide required either dose reduction or treatment cessation due to CIPN. While several patients have experienced coasting (the delayed emergence of CIPN symptoms after drug treatment), 50% experienced an improvement in electrophysiological abnormalities with the gradual reduction in thalidomide dose (Priolo et al., 2008; Lazzerini et al., 2013; Lazzerini et al., 2015).

## 5 Clinical manifestations of pediatric chemotherapy-induced peripheral neuropathy

Pediatric CIPN is often characterized by a combination of sensory, motor, and autonomic symptoms of different intensities (Table 3). CIPN symptoms interfere with the quality of life of pediatric patients and cancer survivors, and, ultimately, with adherence to optimal clinical protocols, as dose reduction is often the only effective strategy to control CIPN (Seretny et al., 2014). The severity and types of symptoms depend on the chemotherapy agents used and the cumulative dose; however, patient-patient variability in sensitivity to chemotherapy and, in turn, the development of CIPN is also observed. Additionally, different combinations of agents used within a chemotherapy regimen often have cumulative effects. Sensory symptoms include paresthesia, dysesthesia, hyperalgesia, allodynia, numbness, pain and loss of proprioception. These typically develop first in the feet and hands, presenting as a “stocking and glove” distal distribution, with the longest axons displaying abnormalities first before spreading proximally as the symptoms progress (Bjornard et al., 2018). In severe instances, these symptoms may result in a loss of sensory perception (Seretny et al., 2014). The occurrence of motor symptoms is less frequent, and include gait abnormalities, balance deficits, fine motor disability and muscle weakness (Kandula et al., 2018). Similarly, autonomic symptoms do not occur as often as sensory symptoms and consist of diarrhea, constipation, urinary retention, incontinence, paralytic ileus, and orthostatic hypotension. The severity of CIPN symptoms is proportional to the cumulative dose of chemotherapy administered and may also develop weeks or months after the completion of treatment (Maestri et al., 2005). Typically, oxaliplatin and paclitaxel have been observed to result in acute CIPN within hours after infusion (Argyriou et al., 2013a). Additionally, some patients treated with chemotherapy drugs, such as oxaliplatin, may experience the worsening of symptoms post-treatment (Park et al., 2013).

**TABLE 3:** Sensory, motor and autonomic symptoms of pediatric CIPN.

Drug	Sensory	Motor	Autonomic	References
Vincristine	Paresthesia, numbness, tingling, loss of sensory discrimination	Upper and lower extremities weakness, wrist- or footdrop, gait abnormalities, balance deficits, fine motor disability, deteriorated deep tendon reflexes, muscle cramps	Constipation, urinary retention, incontinence, paralytic ileus, orthostatic hypotension	Arzanian et al. (2009), Anghelescu et al. (2011), Purser et al. (2014), Courtemanche et al. (2015b), Lavoie Smith et al. (2015), Kerckhove et al. (2017), Bjornard et al. (2018)
Oxaliplatin	Paresthesia and dysesthesia of the hands, feet and perioral region, acute cold hyperesthesia, chronic peripheral hypoesthesia/dysesthesia	Tetanic spasms, fasciculations, prolonged muscular contractions	Constipation, diarrhea	Spunt et al. (2007b), Geoerger et al. (2008), Beaty et al. (2010), Geoerger et al. (2011), Robinson et al. (2016)
Cisplatin	Paresthesia, tingling in the extremities, numbness, mechanical and thermal hyperalgesia, loss of vibration sense and taste	Weakness, tremor	Constipation, diarrhea	Kerckhove et al. (2017), Bjornard et al. (2018)
Carboplatin	Paresthesia	Gait difficulties, ataxia	Constipation, diarrhea	Bjornard et al. (2018)
Paclitaxel	Paresthesia, dysesthesia, numbness, burning pain in a glove-and-stocking distribution	Distal weakness, muscle cramps, muscle aches	Diarrhea, arrhythmias, orthostatic hypotension	Hayashi et al. (2003), Horton et al. (2008), Kerckhove et al. (2017), Bjornard et al. (2018)
Docetaxel	Numbness and tingling in fingers/toes, loss of pinprick sensation and altered reflexes, cold allodynia	Distal weakness, muscle cramps, muscle aches	Diarrhea, arrhythmias, orthostatic hypotension	Kerckhove et al. (2017), Bjornard et al. (2018), Tamburin et al. (2019)
Ixabepilone	Paresthesia, dysesthesia, numbness and pain in the hands and feet	Mild motor impairment with muscle weakness such as foot drop, difficulty in climbing stairs	Limited data available	Widemann et al. (2009), Jacobs et al. (2010), Vahdat et al. (2012), Tamburin et al. (2019)
Bortezomib	Distal sensory loss to all modalities involving the suppression of deep tendon reflexes	Limited data	Limited data available, diarrhea	Blaney et al. (2004), Kerckhove et al. (2017), Bjornard et al. (2018)
Thalidomide	Tingling or painful paresthesia, numbness in the lower limbs	Mild motor impairment	Constipation, diarrhea	Smith et al. (2008), Fleming et al. (2005), Bramuzzo et al. (2017)

Vincristine-induced neurotoxicity occurs during the early stages of treatment, typically within the first month (Kandula et al., 2016). Vincristine-induced motor neuropathy is more prominent in children compared to adults, and manifests as ataxia, foot drop, muscle weakness and gait abnormalities that can be symmetric or asymmetric (Arzanian et al., 2009; Purser et al., 2014; Courtemanche et al., 2015b). Sensory symptoms include paresthesia and dysesthesia, and autonomic symptoms often include constipation, urinary retention, and orthostatic hypotension (Arzanian et al., 2009; Anghelescu et al., 2011; Purser et al., 2014; Courtemanche et al., 2015b). Vincristine also causes cranial neuropathies and a decrease of deep tendon reflexes that manifests as ptosis, hoarse voice, and extraocular eye movement (Lavoie Smith et al., 2015; Kerckhove et al., 2017; Bjornard et al., 2018). Vindesine, a second generation vinca alkaloid, has greater toxicity compared to vincristine, limiting its clinical use (Vats et al., 1992). On the other hand, vinorelbine (third generation) is less neurotoxic than vincristine, possibly due to its decreased capacity to bind to axonal microtubules (Binet et al., 1990).

Oxaliplatin is a third-generation platinum derivative that causes acute and chronic neuropathy. Phase I and II studies in pediatric patients have shown that oxaliplatin causes neurotoxicity that manifested as cold dysesthesia, laryngopharyngeal and limb paresthesia and dysesthesia, muscle cramps and jaw pain. Additionally, oxaliplatin causes diarrhea and constipation (Spunt et al., 2007b; Geoerger et al., 2008; Beaty et al., 2010; Geoerger et al., 2011). Cisplatin is commonly used in combination with vincristine and existing evidence is derived from case reports which proves establishing the incidence and clinical symptoms of peripheral neuropathy in pediatric patients difficult. However, cisplatin causes paresthesia, tingling in the extremities, numbness, mechanical and thermal hyperalgesia, loss of vibration sense and taste, weakness, tremor, constipation, and diarrhea (Kandula et al., 2016).

Paclitaxel causes dose limiting neuropathy that occurs early, within 1 week of the infusion, and is characterized predominantly by sensory symptoms, such as tingling, numbness and burning pain in a “stocking and glove” distribution. Myalgia and gastrointestinal disturbances are also common (Hayashi et al., 2003; Horton et al., 2008; Kerckhove et al., 2017; Bjornard et al., 2018). Adult patients who were administered large cumulative doses have also been observed to exhibit motor symptoms (Freilich et al., 1996), however, such doses are rarely used in pediatric practice (Kandula et al., 2016).

There is limited data describing the development of CIPN symptoms following ixabepilone and bortezomib treatment in the pediatric populations. However, in a phase I study of ixabepilone in children and adolescents with refractory solid tumors, the neuropathy reported was mostly mild, characterized predominantly by sensory symptoms (Widemann et al., 2009; Jacobs et al., 2010). Bortezomib causes sensory neuropathy characterized by sensory loss and suppression of deep tendon reflexes that appears to be more common in adults than in children (Blaney et al., 2004; Messinger et al., 2012; Kerckhove et al., 2017; Bjornard et al., 2018).

Thalidomide causes sensory, motor and autonomic neuropathy that is characterized by paresthesia, numbness, mild motor impairment and constipation (Smith et al., 2008). Thalidomide-induced neuropathy in children is dose-dependent and associated with proximal weakness. Reduction of thalidomide dose leads to improvement of electrophysiological abnormalities (Lazzerini et al., 2015), however, thalidomide neuropathy may also progress after discontinuation of the treatment (Fleming et al., 2005; Bramuzzo et al., 2017).

## 6 Assessment and diagnostic tools of pediatric chemotherapy-induced peripheral neuropathy

Assessment and diagnosis of CIPN among pediatric cancer patients and survivors can be challenging. Children generally are either unable, or find it difficult, to recognize and describe symptoms associated with CIPN. Additionally, the signs and symptoms of CIPN experienced by individuals can be highly variable, and often change over the course of treatment as well as the transition from the pediatric, to adolescent, and adult years. To date, there have been no systematic studies that have specifically examined the temporal course of CIPN symptoms over the course of treatment, nor specifically compared these between different age groups. Accordingly, further research will be needed that integrates factors such as individual susceptibility to CIPN, age, time- and chemotherapy-related factors to determine the most suitable methods for diagnosis and assessment of CIPN for different chemotherapeutics and age groups.

Nevertheless, a range of screening instruments have been developed to diagnose and assess the severity of CIPN symptoms in clinical settings, which can be divided into three major groups: subjective assessments, objective assessments and combined subjective and objective assessments (Table 4). However, there are currently no standardized clinical guidelines for the use of these tools and most of the screening tools discussed below have not been specifically validated in pediatric or adolescent populations.

**TABLE 4:** Tools used for the assessment and diagnosis of pediatric CIPN.

	Instrument	Description	References
Subjective Assessments	Wong-Baker FACES pain scale	Cartoon drawings of faces displaying different levels of pain	Wong and Baker, (1988)
		Most preferred pain rating scale by childrem	
	Pediatric quality of life inventory generic core scales (PedsQL)	Patient-Reported Outcome (PRO) Measures	Varni et al. (2002)
		Assessment of health-related quality of life in children and adolescents with good internal consistency and reliability	
		Includes multidimensional fatigue scale examining general fatigue, sleep/rest fatigue and cognitive fatigue	
	Pediatric outcomes data collection instrument (PODCI)	Patient-Reported Outcome (PRO) Measures	Allen et al. (2008)
		Focus on function and quality of life of the child	
	European organization for research and treatment of cancer (EORTC) chemotherapy-induced peripheral neuropathy questionnaire (CIPN20) and functional assessment of cancer (FACT-G)	Patient-Reported Outcome (PRO) Measures	Postma et al. (2005), Kandula et al. (2018)
		Includes 20-item questionnaire that provides assessment of CIPN-related symptoms and functional limitations of patients treated with chemotherapy	
	The patient-reported outcomes version of the common terminology criteria for adverse events (PRO-CTCAE)	The CTCAE incorporates a library of 790 adverse effects which are graded using an ordinal severity scale	Dueck et al. (2015), Postma et al. (1998)
		Used to assess adverse effects, no specificity for CIPN	
	Modified balis pediatric scale	A children-specific, modified version of CTCAE	Lavoie Smith et al. (2015)
Objective Assessments	Nerve conduction studies	Surface electrodes are used to measure nerve conduction velocity	Ryan et al. (2019)
		Also for children <5 year of age	
	Electromyography (EMG)	EMG is used to asses muscle activity	Kneis et al. (2016), Wright et al. (2017)
		A needle electrode is inserted directly into the muscle group and records the electrical activity	
	Current perception threshold (CPT)	Transcutaneous electrical stimulator used to assess pain, delivering sinusoidal electrical stimuli at different frequencies	Gaudreault et al. (2015)
		Patients are assessed to the degree of pain of the stimulus through forced choice questions	
	Tactile perception threshold (TPT)	Quantitatively measures peripheral sensory nerve function through tactile sensation	Ackerley et al. (2014)
	Vibration perception threshold (VPT)	Quantitatively measures large sensory nerve fibers through vibration	Hilz et al. (1998)
	Movement assessment battery for children (MABC)	Diagnostic tool for identification of impairments in motor performance of children and adolescents 3–16 years of age	Brown and Lalor, (2009)
Combined subjective and objective assessments	Pediatric–modified total neuropathy scale (ped-m TNS)	Assessment of nerve function in children (5–18 years old) and screening for sensory, motor, and autonomic symptoms	Verstappen et al. (2005), Gilchrist et al. (2009), Gilchrist and Tanner, (2013)
		This scale draws upon the Total Neuropathy Scale (TNS) which was initially developed to assess peripheral neuropathy among adults	
	Total neuropathy scale–pediatric version (TNS-PV)	Developed in response to the high impact of vincristine-induced neuropathy in children	Lavoie Smith et al. (2013), Lavoie Smith et al. (2015)
		Screens for chemotherapy associated sensory, motor and autonomic symptoms	
	Neurological exam	Includes assessment of subjective symptoms and objective/physical exam	Haryani et al. (2017)

Subjective assessments used for the diagnosis or assessment of CIPN include quality of life (QoL) questionnaires and pain/neuropathy scales, such as the Pediatric Quality of Life Inventory and Wong-Baker FACES pain scale (for full list see Table 4). Most of these questionnaires and scales are Patient-Reported Outcome (PRO) measures that assess patients’ individual perception of their own health and provide assessment of quality of life, CIPN-related symptoms, and functional limitations of patients. The majority of these PRO measures lack specificity for CIPN, and the individual perception of pain and overall health may vary between patients. Only the European Organization for Research and Treatment of Cancer (EORTC) QLQ-CIPN20 and Functional Assessment of Cancer (FACT-G) has been specifically developed to assess the impact of CIPN and includes the assessment of QoL *via* a 20-item questionnaire assessing CIPN symptoms and functional impairment (Postma et al., 2005). Altogether, the advantage of these scales is the practicality and low cost. However, these subjective tests may not be suitable for very young children, where evaluation of QoL or PRO may only be possible through parents or caregivers, requiring the use of objective assessments such as nerve conduction studies or electromyography.

Electrophysiological tests are objective assessments used for diagnosis or assessment of CIPN. These tests are routinely used to assess the electrophysiological abnormalities associated among patients with CIPN and consist of nerve conduction studies, electromyography (EMG), current perception threshold (CPT) and tactile perception threshold (TPT) tests, to name a few (for full list see Table 4). During these electrophysiological tests, an electrical stimulus is applied to specific nerves or muscles to assess, for example, the sensory nerve action potential (SNAP), compound motor action potential (CMAP), latency and conduction velocities. The quantitative findings are then compared with age-specific norms (Liveson and Ma, 1999). A variation is the vibration perception threshold (VPT) test, which uses vibration (e.g., via a tuning fork) to stimulate sensory neurons (Hilz et al., 1998). Several new electrophysiological assessments for CIPN have been developed but are not yet routinely integrated into clinical practice. For example, the tactile pleasantness test examines touch perception across different skin sites (Ackerley et al., 2014). However, studies have focused on healthy individuals and there is no evidence whether the changes in tactile afferents among patients exposed to neurotoxic chemotherapy agents can be used to diagnose CIPN (Ackerley et al., 2014; Pasqualotto et al., 2020). The advantage of electrophysiological tests for the diagnosis of CIPN is that these tests have a minimal bias and can mostly be performed also on very young children. Keeping the child in a position in which the electrophysiological investigations can be undertaken may be challenging. Additionally, abnormalities detected following chemotherapy such as vincristine, can vary ranging from a purely sensory neuropathy to a sensorimotor neuropathy and very occasionally a pure motor neuropathy with no specific patterns that distinctly characterize vinca alkaloids (Brigo et al., 2012; Courtemanche et al., 2015b; Dudeja et al., 2019). Therefore, studies investigating electrodiagnostic signatures of chemotherapy agents will be necessary.

Another objective test that can be used to assess CIPN in children is the Movement Assessment Battery for Children—Second Edition (MABC-2), which is a functional test that has been developed to assess functional and motor impairment in children (Brown and Lalor, 2009). The MABC-2 has been specifically developed for children and adolescents who are 3–16 years old and includes a performance test to assess motor skills, as well as a checklist. However, the MABC-2 has not been specifically developed to assess CIPN, and further research will be needed to ascertain whether MABC-2 is suitable to assess functional impairments that occur in the setting of CIPN specifically.

Combined subjective and objective assessments are tests that utilize questionnaires and combine these with functional, neurological, or electrophysiological assessments. They include tests such as the Pediatric–modified Total Neuropathy Scale (ped-m TNS) and the Total Neuropathy Scale–Pediatric Version (TNS-PV) (for full list see Table 4). The ped-m TNS is a well validated assessment tool for neuropathy in the pediatric population and specifically screens for neuropathy symptoms in children. The questionnaire assesses sensory, motor, and autonomic neuropathy symptoms and clinical examinations test for light touch, pain and vibration sensation, muscle function and deep tendon reflexes (Gilchrist et al., 2009; Gilchrist and Tanner, 2013). The TNS-PV is a modified version of the ped-m TNS and was specifically developed in response to the high impact of vincristine-induced neuropathy in children and it is well validated in the pediatric population. The TNS-PV additionally assesses responses to temperature, strength, and autonomic and laryngeal neuropathy (Lavoie Smith et al., 2013; Lavoie Smith et al., 2015).

## 7 Risk factors for pediatric chemotherapy-induced peripheral neuropathy

The risk factors for development of CIPN in children include age, ethnicity, genetic susceptibilities, disease factors and treatment factors, to name a few (Table 5). The majority of cases of CIPN in children are attributed to the use of vincristine and platinum-based antineoplastics, as these chemotherapeutic agents are frequently used in pediatric practice (Sałat, 2020). Age may present a risk factor for pediatric CIPN however, the current evidence is conflicting, with studies reporting everything from increased risk of CIPN in younger and older children, to increased risk of CIPN in adults, and some even reporting that there is no evidence for association of age with CIPN (van de Velde et al., 2017; Sałat, 2020; Triarico et al., 2021). On one hand, the incomplete and developing maturation and myelination of the peripheral nervous system in younger children may impose greater risk of developing CIPN following tubulin targeting agents, such as vincristine, which impair the myelination of peripheral nerves (Stadelmann et al., 2019). Younger children also have a faster metabolic rate, which allows them to metabolize chemotherapy drugs faster (de Graaf et al., 1995). This may have two different consequences. Firstly, faster metabolism of the chemotherapy drug may decrease the risk of peripheral nerve damage as the drug is eliminated faster. Secondly, there is little evidence regarding the contribution of chemotherapy metabolites to CIPN, however, this cannot be excluded, and an increase in chemotherapy metabolites may increase the risk of damage to peripheral nerves (Sakurai et al., 2009). Ethnicity imposes greater risk for development of neuropathy following vincristine, which is metabolized via the cytochrome P450 (CYP) 3A4 and CYP3A5 pathways (Dennison et al., 2007). Reduced CYP3A5 activity has been observed in Caucasian children, who are at greater risk to develop vincristine-induced side effects (Dennison et al., 2006; Kishi et al., 2007; Egbelakin et al., 2011; Agrawal et al., 2016). Similarly, allelic variation of the CEP72 gene, which is involved in the formation of microtubules, has been found to play a significant role in development of vincristine-induced neuropathy in children. This variant was more common in Caucasian than African American children, which provides another potential explanation for the interracial differences in the observed incidence of CIPN (Diouf et al., 2015).

**TABLE 5:** Risk factors associated with pediatric chemotherapy-induced peripheral neuropathy.

Risk Factor	Description	References
Age	Younger children may be at greater risk of neuropathies caused by tubulin targeting agents	de Graaf et al. (1995), van de Velde et al. (2017), Stadelmann et al. (2019)
	Younger children have faster metabolism resulting in faster ability to metabolize chemotherapeutic drugs	
Ethnicity	Reduced CYP3A5 activity in Caucasian children	Dennison et al. (2006), Kishi et al. (2007), Egbelakin et al. (2011), Diouf et al. (2015)
	Allelic variation of the *CEP72* gene in Caucasian children	
Sex	No clear evidence in children	van de Velde et al. (2017)
Genetic polymorphisms	Genetic polymorphism in genes *ABCB1*, *ABCC1*, *ACTG1*, *CAPG*, *MAP4*, *SLC5A7*, *TTPA* and *TUBB1*	Ceppi et al. (2014), Wright et al. (2019)
Nutrition	Vitamin B, folate, iron deficiency	Jain et al. (2014), Dudeja et al. (2019), Sajdyk et al. (2020)
	Underweight and overweight	
Pre-existing neuropathies	Charcot-Marie-Tooth or Guillain-Barre syndrome may increase risk for CIPN	Chauvenet et al. (2003), Trobaugh-Lotrario et al. (2003), Bhushan et al. (2015)
Dose	Dose-toxicity relationship for CIPN in children receiving vincristine, thalidomide, docetaxel, paclitaxel and oxaliplatin	Kandula et al. (2016)
Duration of treatment	Treatment duration-toxicity relationship for CIPN in children receiving thalidomide and vincristine	Priolo et al. (2008), Lazzerini et al. (2013), Lazzerini et al. (2015), van de Velde et al. (2017)
Concurrent medications	Concomitant administration of azole antifungal agents, granulocyte colony stimulating factor, nifedipine, cyclosporin, carbamazepine and phenytoin	Weintraub et al. (1996), Hamdy et al. (2012), Moriyama et al. (2012), Teusink et al. (2012), Kandula et al. (2016)

The evidence for sex as a risk factor for CIPN in the pediatric population is unclear (van de Velde et al., 2017), although some studies report that adult female patients are at greater risk of CIPN following paclitaxel treatment compared to adult males (Greenwald et al., 2019). In addition, dietary factors have been associated with risk of CIPN, with vitamin B, folate, iron, and other nutritional deficiencies in pediatric patients with cancer reported to be associated with a greater risk for CIPN (Dudeja et al., 2019). Other studies have found conflicting associations between weight and CIPN, with both underweight and overweight children reported as having an increased risk for CIPN (Jain et al., 2014; Dudeja et al., 2019; Sajdyk et al., 2020). From these studies it is unclear what role diet and/or sex may have in the development of CIPN.

Genetic polymorphism in the genes *ABCB1*, *ABCC1*, *ACTG1*, *CAPG*, *MAP4*, *SLC5A7*, *TTPA* and *TUBB1* could modulate the risk of developing neuropathy in children following vincristine treatment (Ceppi et al., 2014; Wright et al., 2019). The ABCB1 and ABCC1 genes encode for efflux transporter proteins and mutations in which lead to intracellular accumulation of vincristine (Ceppi et al., 2014; Wright et al., 2019). ACTG1, CAPG, MAP4 and TUBB1 encode for genes related to actin or microtubule expression and/or function. The SLC5A7 gene encodes for a choline transporter involved in the synthesis of acetylcholine in cholinergic neurons and was implicated in pathology of inherited neuropathies (Wright et al., 2019). The TTPA gene encodes the α-tocopherol transfer protein that regulates vitamin E levels (Wright et al., 2019). Although studies show that the mutations in those genes modulate the risk to develop neuropathy following vincristine to some degree, additional sufficiently powered studies may be required to validate the association between these genetic polymorphisms and pediatric peripheral neuropathy.

There is some conflicting evidence that pre-existing neuropathies, such as the Charcot-Marie-Tooth or Guillain-Barre syndrome, increase the severity of CIPN (Chauvenet et al., 2003; Bhushan et al., 2015). For example, Charcot-Marie-Tooth is often unrecognized in children, and it is often diagnosed after children treated with chemotherapy experience severe sensory and motor neuropathy symptoms (Trobaugh-Lotrario et al., 2003). Additionally, patients with pre-existing neuropathy are rarely included in clinical trials, hampering the identification of pre-existing neuropathy as risk factor for CIPN.

Treatment factors, such as cumulative dose, the duration of the chemotherapy treatment and concomitant administration of other medications such as triazole or imidazole have been associated with higher frequency and severity of CIPN following chemotherapy treatment (Table 5). Kandula et al. reported evidence supporting a dose-toxicity relationship for CIPN in children receiving vincristine, thalidomide, docetaxel, paclitaxel and oxaliplatin (Kandula et al., 2016). However, the dose-dependence of CIPN development appears to be specific to the chemotherapy agent or combination regimen used. The duration of the chemotherapy treatment is another possible risk factor for CIPN. Higher doses of thalidomide over a short period of time were associated with lower risk for CIPN compared to regimens that involved a lower dose of thalidomide over a longer period (Priolo et al., 2008; Lazzerini et al., 2013; Lazzerini et al., 2015). In contrast, for vincristine a bolus administration over 1–5 min led to higher distribution rate of vincristine within central and peripheral compartments compared to circulating vincristine, causing more severe CIPN (van de Velde et al., 2017). Therefore, a prolonged vincristine infusion could be implemented as strategy for preventing CIPN. Additionally, some evidence shows that the risk of developing CIPN was higher at the initial phase of the chemotherapy compared to later stages of chemotherapy (Langholz et al., 2011). The dose of chemotherapy is often reduced early in the treatment due to patients experiencing CIPN symptoms, which may perhaps explain the higher risk for CIPN in the initial stage of treatment. The use of concomitant medications, such as azole antifungal agents, with chemotherapy has been associated with increased frequency and severity of CIPN. Imidazole and triazole inhibit the CYP3A4, consequently inhibiting vincristine metabolism (Hamdy et al., 2012; Moriyama et al., 2012; Teusink et al., 2012; Kandula et al., 2016). Of note, granulocyte colony stimulating factor is often used to support the mobilization of hematopoietic stem cells after bone marrow transplantation or following high-dose multi-agent chemotherapy in children, although its use has been associated with higher risk for CIPN (Weintraub et al., 1996; Packer et al., 2006). Nifedipine, cyclosporin, carbamazepine and phenytoin may modulate pharmacokinetics of chemotherapy agents and, in turn, the incidence of CIPN, although this evidence stems from case reports (Kandula et al., 2016).

## 8 Pathophysiological processes involved in chemotherapy-induced peripheral neuropathy

**FIGURE 2:**
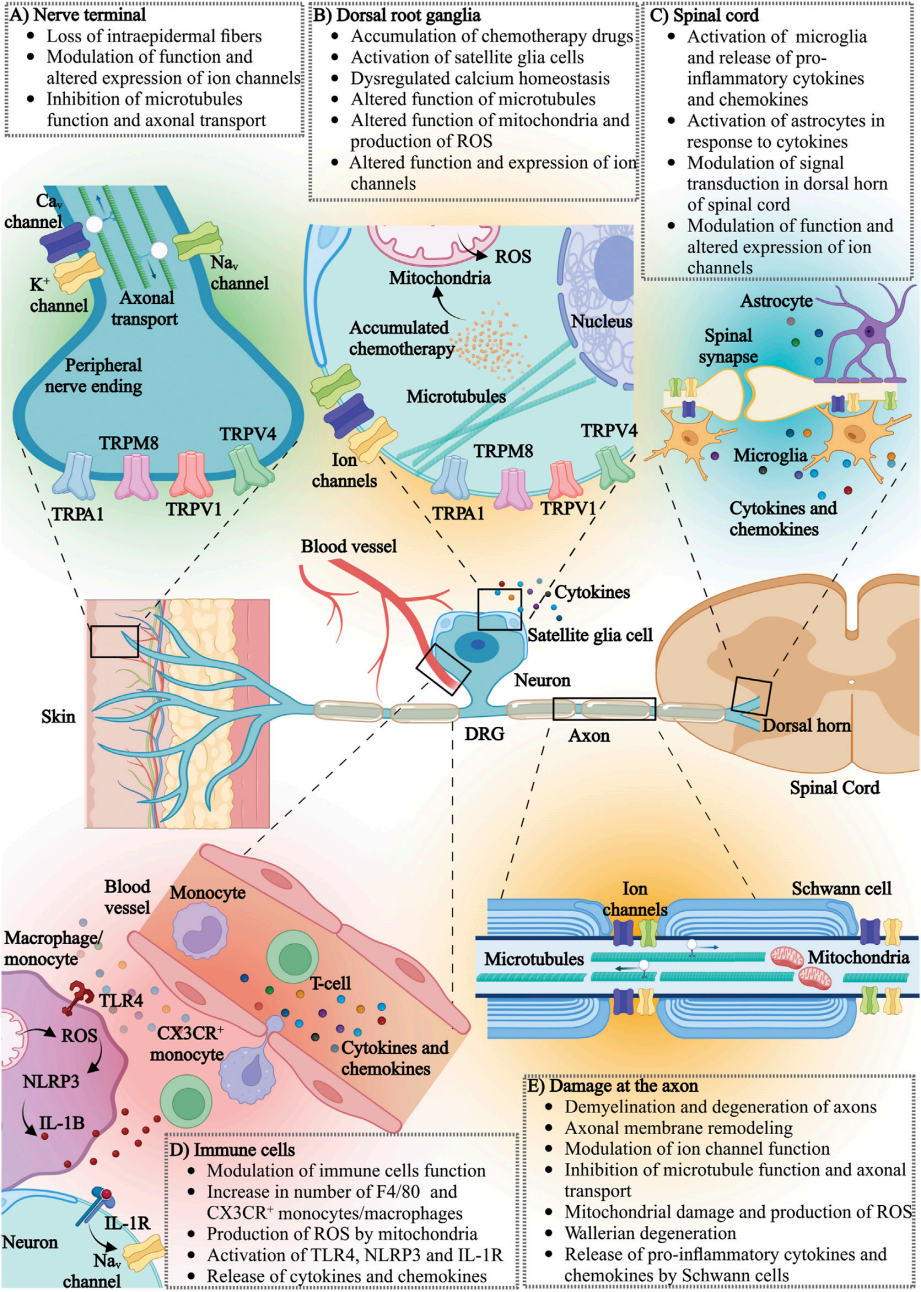
The effects of chemotherapy on various components of the nervous and immune system: **(A)** nerve terminal, **(B)** dorsal root ganglia (DRG), **(C)** spinal cord, **(D)** immune cells and **(E)** neuronal axons. Ca_V_, voltage-gated calcium channel; K^+^, potassium channel; Na_V_, voltage-gated sodium channel; TRPA1, transient receptor potential (TRP) ankyrin 1; TRPM8, transient receptor potential cation channel subfamily melastatin member 8; TRPV1, transient receptor potential vanilloid-type 1; TRPV4, transient receptor potential vanilloid-type 4; ROS, reactive oxygen species; TLR4, toll-like receptor 4; NLRP3, NOD-, LRR- and pyrin domain-containing protein 3; IL-1B, interleukin-1 beta; IL-1R, interleukin-1 receptor; CX3CR, CX3C chemokine receptor 1.

The pathological mechanisms involved in pediatric CIPN are poorly understood. One major obstacle in understanding the pathology of pediatric CIPN is that most of the mechanistic evidence stems from studies that utilize adult rodent models or adult patients. Evidence from these studies clearly demonstrates that CIPN involves complex interactions between peripheral and central neurons and microenvironmental niches, including immune cells and neuronal accessory cells (Ji et al., 2016). The cellular and molecular functions of these cells cannot be assumed to be identical between juveniles and adults, and hence it is conceivable that the pathophysiological mechanisms contributing to pediatric CIPN differ from those in adults. Specifically, it is known that the reactivity and composition of the innate and adaptive immune system, myelination of peripheral nerves, the expression and function of ion channels involved in action potential generation and propagation in neurons, as well as CNS neuroplasticity differs in children compared to adults (Loizzo et al., 2009; Kollmann et al., 2012; Simon et al., 2015; Stadelmann et al., 2019; Strickland et al., 2019; Bhatt et al., 2020). Although appropriate juvenile models are thus arguably critical for comparison of the pathophysiological processes contributing to CIPN in adult and pediatric populations, these are currently lacking, possibly due to ethical and practical reasons. For these reasons, it is not clear to what extent evidence obtained in adult humans or rodent models can be applied to pediatric CIPN. Accordingly, in the following sections knowledge regarding the pathophysiological mechanisms underlying CIPN—including axon degeneration, activation of the immune system and neuroinflammation, mitochondrial disfunction, dysregulated calcium homeostasis, altered neuronal hyperexcitability and changes in ion channel activity (Figure 2) will be discussed with the caveat that these insights were obtained in adults, and may not necessarily be identical in pediatric patients.

### 8.1 Neuronal damage

Chemotherapy agents are known to cause microscopic changes to peripheral nerves, such as myelin loss, changes to the cytoskeleton and axon degeneration, that can be visualized using standard histology and immunohistochemistry methods and are possibly specific to the chemotherapy agent (Figure 2). For example, platinum derivates and tubulin targeting agents accumulate in dorsal root ganglia neurons (Cavaletti et al., 1997; Cavaletti et al., 2000; Liu et al., 2013; Sprowl et al., 2013; Wozniak et al., 2016). Accumulated chemotherapeutics in the cell body of a peripheral neuron may interfere with cellular metabolism, axoplasmic transport and function of mitochondria, ultimately leading to cell death. While the mechanisms of accumulation of chemotherapy agents in dorsal root ganglion neurons is unknown, some evidence shows that the accumulation of platinum derivates may be due to high expression of specific membrane transporters, such as the organic anion-transporting polypeptides (OATPs), and the organic cation transporter (OCT) that are responsible for platinum transport (Gregg et al., 1992; Screnci et al., 2000; Fujita et al., 2019; Huang et al., 2020).

Studies in animals and humans have also shown that the prolonged use of chemotherapeutic agents such as vincristine, oxaliplatin, cisplatin, carboplatin, paclitaxel, bortezomib and ixabepilone can result in degeneration of large, myelinated axons and the loss of intraepidermal nerve fibers (Sahenk et al., 1994; Neijt et al., 2000; Parmar et al., 2003; Boyette-Davis et al., 2013; Boehmerle et al., 2014; Ebenezer et al., 2014; Yamamoto et al., 2015a; Geisler et al., 2016; Turkiew et al., 2017; Malacrida et al., 2019; Ogihara et al., 2019). The extent of neuronal damage has been correlated with different degrees of neuropathy severity, but the precise underlying mechanisms by which demyelination and degeneration of peripheral nerves contribute to CIPN remain unclear (Colvin, 2019; Malacrida et al., 2019). These structural changes in the peripheral nervous system are probably a reflection of chronic CIPN and long-term damage caused by chemotherapy. For example, vincristine and paclitaxel induced CIPN is often associated with loss of intraepidermal nerve fibers (IENF), which are thinly myelinated Aδ fibers and unmyelinated C fibers, that transmit temperature and pain signals (Siau et al., 2006; Beiswenger et al., 2008; Myers et al., 2013; Geisler et al., 2016; Mangus et al., 2020). However, mechanical allodynia in rodents following vincristine develops within 24 h of injection with no apparent changes in IENF quantity during the first week (Starobova et al., 2021). Chemotherapy-induced neuronal damage is likely specific to the chemotherapy agent and its specific mode of action. Vincristine and paclitaxel affect large, myelinated fibers, while cisplatin damages all types of myelinated fibers, and bortezomib affects small, unmyelinated fibers (Boehmerle et al., 2014; Gornstein and Schwarz, 2014). The specific mechanisms of neuronal demyelination of large neuronal fibers caused by vincristine and paclitaxel are unclear, however the disruption of microtubule function and the subsequent impairment of retrograde and anterograde axonal transport of essential cellular components may play a role (Sahenk et al., 1994; Siau et al., 2006; Boehmerle et al., 2014; Gornstein and Schwarz, 2017). The degeneration of distal nerve segments and axonal membrane remodeling is known as Wallerian degeneration (Yang et al., 2009; Fukuda et al., 2017). Recent pre-clinical studies have suggested that Wallerian-like degeneration is driven by sterile alpha and toll/interleukin-1 receptor motif-containing 1 (SARM1) (Bosanac et al., 2021). SARM1 is an inducible NAD^+^ hydrolase that triggers the loss of axons and neuronal cell death, and both SARM1 and its intrinsic NADase activity are required for injury-induced axon degeneration (Essuman et al., 2017; Bosanac et al., 2021; Ko et al., 2021). Genetic deletion of *SARM1* protected rodents treated with paclitaxel from distal degeneration of small fibers (Turkiew et al., 2017). Additionally, the genetic deletion of *SARM1* was demonstrated to prevent vincristine-induced neuropathy and bortezomib-induced axon destruction (Geisler et al., 2019).

### 8.2 Molecular mechanisms of chemotherapy-induced peripheral neuropathy

#### 8.2.1 Activation of the immune system and neuroinflammation

A traditional view has been that chemotherapy agents have predominantly immunosuppressive properties due to their effects on rapidly dividing cells, including immune cells (Zitvogel et al., 2008). This is certainly evident from the profound myelosuppression that can occur clinically following chemotherapy treatment. Recent studies show that chemotherapy agents can also activate the innate and adaptive immune system, presenting a double-edged sword (Byrd-Leifer et al., 2001; Zitvogel et al., 2008; Starobova et al., 2020a; Starobova et al., 2021). On one hand, activation of inflammatory cascades may have a positive impact on patient recovery and response of tumors to chemotherapy treatment (Zitvogel et al., 2008). On the other hand, dysregulation of neuro-inflammatory processes in the dorsal root ganglia, axons, and spinal cord, including the dysregulation of cytokine and chemokine levels, increased presence and activation of immune cells, and activation of neuronal accessory cells, has been associated with CIPN pathology. Specifically, increased levels of pro-inflammatory cytokines such as interleukin-1β (IL-1β), interleukin-6 (IL-6), interleukin-8 (IL-8), tumor necrosis factor α (TNFα), interferon γ (IFN-γ) and chemokines, such as CCL2, CXCL12, CCL11, CCL3, and CCL4 have been observed following vincristine, oxaliplatin, cisplatin and paclitaxel administration in human, rodent and *in vitro* studies (White et al., 1998; Wang et al., 2012; Makker et al., 2017; Brandolini et al., 2019a; Brandolini et al., 2019b; Kleckner et al., 2021). Additionally, a decline in the expression of anti-inflammatory cytokines (IL-10 and IL-4) was observed (Makker et al., 2017; Brandolini et al., 2019a; Brandolini et al., 2019b). The origin of these cytokines and chemokines can be attributed to the increased activation of immune cells and neuronal accessory cells. For example, an increase in the number of immune cells, such as macrophages, in the proximity of peripheral nerves has been implicated in CIPN pathology (Figure 2). Specifically, the number of F4-80^+^ cells, which include monocytes and macrophages, was increased in dorsal root ganglia and sciatic nerve following vincristine and paclitaxel treatment, correlating with CIPN symptoms development (Peters et al., 2007; Old et al., 2014; Starobova et al., 2021). Conversely, depletion of these cells using liposomal clodronate alleviates CIPN symptoms in rodents (Peters et al., 2007; Old et al., 2014; Starobova et al., 2021). Activation of sensory neuron TRPA1 channels downstream of reactive oxygen species produced by these CX3CR+ macrophages likely accounts for sensory symptoms associated with CIPN, at least in part (Old et al., 2014; Montague et al., 2018). In contrast, the contribution of neutrophils to the development of CIPN is less clear: an increase in neutrophils in the blood of breast cancer patients receiving paclitaxel has been associated with CIPN, although no increase in the number of neutrophils was observed in the DRGs of rodents following treatment with paclitaxel (Ruiz-Medina et al., 2013). Similarly, oxaliplatin treatment did not increase DRG neutrophil numbers, and depletion of neutrophils in rodents receiving vincristine did not reverse CIPN symptoms (Makker et al., 2017; Starobova et al., 2021).

Central and peripheral neuronal accessory cells such as the central microglia, astrocytes, satellite glial cells and Schwann cells can also release pro-inflammatory cytokines and chemokines in the neuronal microenvironment and contribute to the development of CIPN, in particular in paclitaxel-induced neuropathy (Cavaletti et al., 1995; Peters et al., 2007; Kiya et al., 2011; Burgos et al., 2012; Ruiz-Medina et al., 2013; Robinson et al., 2014) (Figure 2). The role of T-cells in CIPN is still unclear, although some studies report an increase in circulating CD4^+^ and CD8^+^ T-cells following paclitaxel or oxaliplatin treatment, with the presence of CD8^+^ T-cells in dorsal root ganglia appearing crucial for the resolution of CIPN symptoms (Zhang et al., 2008; Krukowski et al., 2016; Makker et al., 2017).

The molecular mechanisms contribution to activation of the innate immune system and neuronal accessory cells following chemotherapy treatment are still unclear, although activation of specific pro-inflammatory receptors may play a role. For example, the Nod-Like-Receptor 3 (NLRP3) inflammasome is predominantly expressed by macrophages and neutrophils and to some extent by dendritic cells, microglia and possibly by dorsal root ganglia (Guarda et al., 2011; Gustin et al., 2015; Fann et al., 2018). NLRP3 was previously implicated in the pathology of painful conditions and is activated in two steps (Starobova et al., 2020b). The first step includes the priming of the NLRP3 inflammasomes via the activation of Toll-like receptors such as the Toll-like receptor 4 (TLR4) leading to NF-kB-dependent expression of NLRP3, pro-IL-1β and pro-IL-18. The second step is the activation of the NLRP3 inflammasome by so-called DAMPs (danger-associated molecular patterns) and PAMPs (pathogen-associated molecular patterns), leading to assembly of a large inflammasome complex that provides an activation platform for caspase 1 and subsequent cleavage of pro-IL-1β and pro-IL-18 into their active forms (Bauernfeind et al., 2009; Tschopp and Schroder, 2010). With regards to chemotherapeutics, tubulin targeting agents such as paclitaxel and vincristine were shown to either prime NLRP3 via TLR4, or to indirectly activate NLRP3 leading to release of IL-1β. IL-1β-mediated sensory neuron sensitization via modulation of ion channel function could, in turn, be prevented by genetic deletion or direct inhibition of TLR4, NLRP3 or IL-1R in rodents treated with vincristine, paclitaxel or oxaliplatin (Byrd-Leifer et al., 2001; Son et al., 2019; Starobova et al., 2019; Starobova et al., 2021).

How chemotherapeutics activate NLRP3 is unclear. One possible activation pathway could be via increased production of reactive oxygen species caused by mitochondrial disfunction (Cruz et al., 2007; Tschopp and Schroder, 2010). Mitochondria are cellular organelles that control the transformation of energy and production of adenosine triphosphate (ATP) and are the main source and targets of reactive oxygen species (ROS). Various chemotherapeutics damage neuronal and non-neuronal mitochondria, increasing the production of ROS (Sangeetha et al., 1990; Look and Musch, 1994; Weijl et al., 1998; McDonald and Windebank, 2002; Canta et al., 2015). Although the mechanisms leading to mitochondrial damage are likely specific to the chemotherapy agent, the underlying mechanisms are poorly understood (Doyle et al., 2012; Griffiths and Flatters, 2015) . ROS produced in small amounts in healthy tissue carry out important signaling functions; however, increased ROS production may damage intracellular biomolecules, activate NLRP3 and lead to increased release of pro-inflammatory cytokines (Slater, 1984; Stadtman et al., 2003; Valko et al., 2005). These processes may further damage mitochondria, amplifying ROS production and neuronal damage including demyelination of axons and disruption of the cytoskeleton (Zheng et al., 2011).

#### 8.2.2 Dysregulated calcium homeostasis

As calcium (Ca^2+^) is a ubiquitous signaling molecule and particularly important for function of sensory neurons, it is perhaps not surprising that evidence of dysregulated Ca^2+^ homeostasis has been reported for several chemotherapeutics, including oxaliplatin, cisplatin, paclitaxel, bortezomib and vincristine (Tomaszewski and Busselberg, 2007; Chen et al., 2017; Leo et al., 2017; Li et al., 2017; Chine et al., 2019; Tomita et al., 2019). As discussed below, dysregulation of calcium homeostasis includes direct changes to the expression of Ca^2+^ channel subunits, but also global changes to intracellular Ca^2+^ homeostasis, in particular mitochondrial Ca^2+^ levels which in turn may be associated with altered mitochondrial function (Figure 2). A direct link to Ca^2+^ sequestration has been proposed for oxaliplatin, whose metabolites includes a Ca^2+−^chelating oxalate moiety. Indeed, local oxalate injection induces spontaneous nocifensive behaviors and mechanical allodynia in rodents, although the pathognomonic cold allodynia typically seen following oxaliplatin treatment could not be recapitulated consistently (Sakurai et al., 2009; Deuis et al., 2013). Nonetheless, administration of Ca^2+^/Mg^2+^ infusions prior to oxaliplatin has shown some positive effects on CIPN development in clinical trials (Deuis et al., 2013; Canta et al., 2015; Jordan et al., 2016).

#### 8.2.3 Altered neuronal excitability

Chemotherapeutic agents associated with development of sensory peripheral neuropathy, such as vincristine, oxaliplatin, cisplatin, paclitaxel and bortezomib, have been observed to alter peripheral nerve excitability. Changes to the expression and function of ion channels such as voltage-gated sodium (Na_V_), voltage-gated potassium (K_V_), voltage-gated calcium (Ca_V_) and transient receptor potential family (TRP) channels are believed to contribute substantially to the cascade of events that lead to neuronal hyperexcitability (Figure 2).

Na_v_ channels are essential for nociceptive signal transduction and propagation, with at least five of the nine mammalian subtypes (Na_V_1.1, 1.6, 1.7, 1.8 and 1.9) being expressed in dorsal root ganglion neurons. Although involvement of tetrodotoxin (TTX)-resistant Na_V_ isoforms, most notably Na_V_1.8 and Na_V_1.9, in CIPN has been suggested (Descoeur et al., 2011; Argyriou et al., 2013b; Lolignier et al., 2015), several lines of evidence implicate TTX-sensitive Na_V_ isoforms as key contributors to enhanced excitability and allodynia. Both oxaliplatin-induced nerve hyperexcitability and allodynia, as well as paclitaxel-induced mechanical and cold allodynia, could be reversed by TTX in mice (Webster et al., 2005; Nieto et al., 2008; Deuis et al., 2013), and some positive effects of subcutaneous TTX on QoL indicators were also reported in a small clinical trial (Goldlust et al., 2021). The Na_V_ subtypes contributing to this effect appear to be agent-specific, with the TTX-sensitive Na_V_1.6 contributing to vincristine-induced mechanical and oxaliplatin-induced cold allodynia (Sittl et al., 2012; Deuis et al., 2013; Chen et al., 2020), while contributions of the TTX-sensitive Na_V_1.7, including increased expression and trafficking, were seen in both rodent and human dorsal root ganglion neurons after administration of paclitaxel (Akin et al., 2021; Goldlust et al., 2021). While further confirmation of similar pathological mechanisms in humans is needed, modulation of Na_V_ channel function remains an attractive strategy for treatment of sensory symptoms associated with CIPN.

Potassium (K^+^) channels regulate the excitability and resting membrane potential of a cell and shape the action potential waveforms in neurons. They are classified into 4 groups, namely the voltage-gated K (K_V_) channels, calcium-activated potassium (K_Ca_) channels, inwardly rectifying potassium channels (Kir) and two-pore domain potassium (K2P) channels. Enhanced neuronal excitability attributable to altered expression and/or function of K^+^ channels has been suggested as a putative pathophysiological mechanism for some chemotherapeutic agents, although the functional heterogeneity of these channels, which can assemble as homo- or heteromultimers, has made dissection of specific subtype contributions comparatively difficult. Evidence of functionally altered K^+^ conductances arose from observations that oxaliplatin treatment leads to broadening of the repolarization phase as well as repetitive firing and afterhyperpolarization (AHP) in isolated rat sciatic nerve (Kagiava et al., 2008). Altered expression of TREK1, TREK2 and TRAAK potassium channels, M-type (KCNQ) channels, K_V_1.1 and K_V_4.3 have all been attributed to treatment with oxaliplatin (Descoeur et al., 2011; Thibault et al., 2012; Pereira et al., 2014; Poupon et al., 2018; Viatchenko-Karpinski et al., 2018). The contribution of K^+^ channels to CIPN induced by other agents is less clear, with upregulation of a range of channel subtypes/genes reported for paclitaxel (Zhang and Dougherty, 2014), but little information is available on the effect of other chemotherapeutics on K^+^ channel expression and function.

Consistent with their well-defined roles in pain signaling, upregulation of both the pore-forming α subunit of Ca_V_2.2 channels, as well as the α_2_δ auxiliary subunit, has been reported in response to cisplatin, paclitaxel and oxaliplatin, but not vincristine (Matsumoto et al., 2006; Xiao et al., 2007; Gauchan et al., 2009; Kawakami et al., 2012; Leo et al., 2017). Similarly, paclitaxel also led to an increase in T-type current amplitude and density in DRG neurons, in conjunction with up-regulation of Ca_V_3.2 protein (Li et al., 2017).

Transient receptor potential (TRP) channels function as stimulus transducers and often respond to thermal or chemical signals—including protons and reactive oxygen species—leading to activation and sensitization of peripheral nerve terminals. The intense interest in the role of TRP channels in various pain states has also extended to CIPN, often with conflicting results that may arise due to species, dose, or other experimental differences. Particular interest in pathognomic cold hypersensitivity induced by oxaliplatin has seen the cold-thermosensors TRPA1 or TRPM8 implicated by several studies (Yamamoto et al., 2015b; Nakagawa and Kaneko, 2017; Chukyo et al., 2018; Rimola et al., 2021; Wu et al., 2021; Marcotti et al., 2022), although others have found no contribution (Deuis et al., 2013). TRPA1 and TRPM8, but also the heat-sensitive TRPV1 and more recently, TRPV4, have also been found to be involved in paclitaxel-, vincristine- and thalidomide-induced neuropathy, where both increased channel expression and analgesic effects from antagonists were reported (Alessandri-Haber et al., 2004; Chen et al., 2011; Materazzi et al., 2012; Hara et al., 2013; Chiba et al., 2017; Boehmerle et al., 2018). The molecular mechanisms leading to altered TRP channel activity in these conditions remain unclear, although it appears likely that oxidative stress may contribute (Alessandri-Haber et al., 2008; Materazzi et al., 2012; Old et al., 2014; De Logu et al., 2020).

## 9 Chemotherapy-induced peripheral neuropathy management

### 9.1 Pharmacological management

The management of pediatric CIPN includes preventative strategies as well as pharmacological and non-pharmacological measures to control symptoms. The future of CIPN prevention and treatment arguably lies in the development of mechanism-based treatment strategies, targeting specific molecular structures and preventing peripheral nerve damage caused by chemotherapy agents. Currently, pharmaceutical agents used for the prevention and/or management of CIPN include supplements, such as calcium, acetyl carnitine, glutamine, and omega 3, and medications, such as amifostine, amitriptyline, venlafaxine, oxcarbazepine, and carbamazepine (Hershman et al., 2014). The benefit of using these agents in children is unclear, as most studies were conducted in adult populations and the benefits for CIPN patients are often inconclusive. Duloxetine is the only drug shown to have moderate effect on CIPN in adults receiving oxaliplatin and paclitaxel and is thus the only treatment recommended for CIPN by The American Society of Clinical Oncology (ASCO) (Hershman et al., 2014; Meng et al., 2019). Carbamazepine and gabapentin are both anticonvulsants that modulate the function of neuronal ion channels, but evidence showing positive effects of these drugs on CIPN is mostly inconclusive due to small cohort sizes and a lack of studies in the pediatric population (von Delius et al., 2007; Eckel et al., 2002; Rao et al., 2007). The use of pyridoxine (Vitamin B6) and pyridostigmine for the prevention of CIPN has shown some benefit. Pyridostigmine is used to enhance decreased gastrointestinal motility in children (Manini et al., 2018; Aydin Koker et al., 2021). Case reports suggest that pyridoxine alleviates vincristine-induced neuropathy in children (Jackson et al., 1986; Muller et al., 2004; Duman et al., 2005; Ngamphaiboon et al., 2010; Aydin Koker et al., 2021). Additionally, various studies performed in adult patient populations have demonstrated that vitamin D deficiency increases the severity of CIPN, while supplementing vitamin D during chemotherapy may improve neuropathy symptoms (Wang et al., 2016; Grim et al., 2017; Jennaro et al., 2020; Yildirim and Cengiz, 2020). However, these findings are limited by small sample sizes and further validation is needed to demonstrate significant clinical utility for vitamin D particularly for pediatric cancer patients (Tofthagen et al., 2022).

Some studies have also explored topical administration of drugs to manage CIPN. Topical gel containing baclofen (10 mg), amitriptyline HCL (40 mg), and ketamine (20 mg) alleviated CIPN symptoms such as cramping and tingling in patients but this outcome was associated with higher systemic toxicity (Barton et al., 2011; Gewandter et al., 2014).

Due to the lack of efficacy of the above-mentioned management strategies for CIPN, in recent years the focus has shifted to delineating the molecular mechanisms underlying CIPN, with the view to identify mechanism-specific treatment targets. As discussed above, CIPN following some chemotherapy agents is driven by neuro-inflammatory processes, suggesting that inhibition of specific immune processes may be an effective treatment strategy. For example, vincristine, ixabepilone and paclitaxel-induced peripheral neuropathies are driven by neuroinflammatory processes, in particular by the release of nerve sensitizing IL-1β. Accordingly, CIPN symptoms were alleviated following the co-administration of IL-1 receptor-targeting agents such as anakinra (Kineret^®^) in *in vivo* rodent models (Kuyrukluyildiz et al., 2016; Starobova et al., 2021). Additionally, Starobova et al. found that anakinra co-administration is safe, with no negative effects on tumor progression or vincristine efficacy in medulloblastoma patient derived xenograft models (Starobova et al., 2021). Similarly, minocycline, a second-generation tetracycline derivative, alleviated CIPN symptoms and histological abnormalities in tissue following vincristine, oxaliplatin and paclitaxel administration in rodent models, likely through inhibition of pro-inflammatory processes (Boyette-Davis and Dougherty, 2011; Boyette-Davis et al., 2011; Starobova et al., 2019). The exact mode of action of minocycline is not known, however it is widely accepted that minocycline inhibits the activation of monocytes and microglia and exerts a range of anti-inflammatory properties (Ledeboer et al., 2005; Huang et al., 2014; Salat et al., 2021). In one clinical study, minocycline decreased overall the pain scores and fatigue in patients treated with paclitaxel (Pachman et al., 2017). Metformin is another anti-inflammatory agent that has been explored as a treatment of CIPN in rodents. Metformin inhibits macrophage activation and decreases the release of pro-inflammatory cytokines, such as TNFα and IL-6 (Huang et al., 2009). Rodents treated with cisplatin or paclitaxel and co-treated with metformin experienced milder CIPN symptoms compared to control. This included the alleviation of mechanical allodynia, numbness, and reduced loss of intraepidermal nerve fibers (Mao-Ying et al., 2014). One clinical study has investigated the benefits of metformin in breast cancer patients treated with paclitaxel and anthracycline. Metformin decreased the incidence of peripheral neuropathy and bone pain but exacerbated gastrointestinal disturbances (Barakat et al., 2022). Erythropoietin is a cytokine produced by the kidney, with several rodent studies demonstrating that erythropoietin improves nerve regeneration, functional nerve recovery and partially prevents intraepidermal fiber loss following cisplatin and docetaxel administration (Bianchi et al., 2006; Bianchi et al., 2007; Cervellini et al., 2010; Yin et al., 2010).

Generally, the use of FDA approved anti-inflammatory drugs such as anakinra, minocycline or metformin for the prevention of CIPN is a strategy that may lead to accelerated translation of preclinical studies into the clinical setting. However, the addition of an anti-inflammatory drug to the chemotherapy regimen may have adverse impacts on tumor progression and chemotherapy efficacy, and high-quality clinical studies addressing these issues as well as efficacy are urgently needed.

### 9.2 Non-pharmacological approaches

There is limited evidence regarding non-pharmacological methods for effectively managing CIPN in children, with most evidence stemming from adults (Table 6). Only a few studies investigating small cohorts of children show improvement in pain perception and balance with non-pharmacological interventions; and many participants had ongoing impairments in distal muscle strength, reduced deep tendon reflexes, and sensory deficits. Given these data, further research in the field of pediatric CIPN is warranted (Casanova-García et al., 2015; Gilchrist and Tanner, 2018). This dearth of high-quality and consistent evidence was also noted in recent clinical practice guidelines, which—as noted above—only support duloxetine as moderately effective for treatment of existing CIPN (Hershman et al., 2014). More recently, Gu et al. reported a 20-year bibliometric analysis highlighting the rise in efforts to study non-pharmacological interventions for CIPN reaching a high point in only the last few years (Gu et al., 2022). Of note, some of the key potential mechanisms underpinning CIPN, including inflammation, may be influenced by exercise, providing a possible mechanistic link (Chung et al., 2022). Other non-pharmacological, complementary, and non-opioid methods that have been investigated for the prevention and treatment of CIPN, such as photobiomodulation therapy, also have anti-inflammatory and neuro-immunological effects (Martins et al., 2016; Kobiela Ketz et al., 2017).

**TABLE 6:** Status of types of non-pharmacological methods to prevent, treat or limit severity of CIPN.

Intervention	Status and potential	References
Exercise	Individualized, multimodal exercise programs that are undertaken during chemotherapy and include balance training and sensorimotor integration have greatest potential to influence CIPN	Duregon et al. (2018), Oh and Kim, 2018), Kanzawa-Lee et al. (2020), Tanay et al. (2021), Brett Whalen et al. (2022), Guo et al. (2022)
	Matters of dose and patient compliance need addressing	
Acupuncture and derivatives	Mixed outcomes using different forms of acupuncture suggest further research in this field is required to determine optimal treatment parameters	Brami et al. (2016), Hsieh et al. (2016), Wong et al. (2016), Derksen et al. (2017), Oh and Kim, (2018), Li et al. (2019), Hao et al. (2020), Chien et al. (2021)
Electrophysical agents	Photobiomodulation, electrical stimulation and thermal therapies show some positive effects on CIPN	Coyne et al. (2013), Argenta et al. (2017), Gewandter et al. (2019), Bandla et al. (2020), Lodewijckx et al. (2020), Oneda et al. (2020), Song et al. (2020), Chitkumarn et al. (2022), Lodewijckx et al. (2022)
	Once mechanism/s of CIPN identified, therapies that target those mechanisms may have greatest potential (e.g., PBM which has a neuroimmune effect)	
Combination therapies	Multimodal preventive and treatment strategies (e.g., exercise ± PBM ± acupuncture) may address different pathophysiological effects of chemotherapies	Schönsteiner et al. (2017), Kurt and Can (2018), Galantino et al. (2019)

Exercise alone or in combination has been reported increasingly in recent years for its effect in preventing or treating CIPN however, the evidence stems from studies in adult patients. Several systematic reviews and meta-analyses show beneficial effects of exercise on QoL and CIPN symptoms, including improved muscle strength and endurance (Duregon et al., 2018; Kanzawa-Lee et al., 2020; Tanay et al., 2021). The most recent literature syntheses suggest that exercise undertaken concurrently with chemotherapy is significantly beneficial in preventing, mitigating, or improving CIPN symptoms and associated sleep disturbances (Brett Whalen et al., 2022). Similar findings were also reported by Guo et al., where significant effects of exercise on QoL and CIPN symptoms, including neuropathic pain, upper and lower limb muscle strength and increased balance performance were reported (Guo et al., 2022). Other studies investigating the benefits of exercise for CIPN management are often inconsistent and report both improvement, or lack of positive effects of exercise on CIPN symptoms (Fernandes and Kumar, 2016; Kleckner et al., 2018; Kneis et al., 2019; McCrary et al., 2019; Dhawan et al., 2020). The disparate nature of the findings may be related to the variety of exercises, exercise protocols and dosing regimens investigated. Additionally, exercise compliance is likely to be another factor that determines whether study participants gain benefits from exercise (Kneis et al., 2019; Müller et al., 2021).

Several recent studies, systematic reviews and meta-analyses have investigated acupuncture and related procedures, such as electro or laser acupuncture, for the management of CIPN (Brami et al., 2016; Greenlee et al., 2016; Hsieh et al., 2016; Wong et al., 2016; Derksen et al., 2017; Oh and Kim, 2018; Hwang et al., 2020; Chien et al., 2021). Despite some evidence that acupuncture may decrease sensory neuron hypersensitization through GABA-ergic, serotoninergic and adrenergic effects (Hao et al., 2020), evidence supporting the benefits of acupuncture for the management of CIPN remains limited.

Electrophysical agents encompass methods that incorporate physical, thermal, magnetic, or electrical energies either applied to the body or used as a medium for biofeedback or diagnosis. One such agent is photobiomodulation (PBM) which has effects at the molecular/genetic, cellular and systems levels. PBM has found some support in recent placebo/sham-controlled studies for reducing and/or controlling neuropathy pain, function and symptoms (Argenta et al., 2017; Lodewijckx et al., 2020; Lodewijckx et al., 2022). Electrical stimulation (eStim) has nerve blocking and endogenous opioid effects, and has been reported to improve pain scores, tingling, numbness, and cramping (Gewandter et al., 2019). However, in a randomized, placebo-controlled study of 72 participants, low-frequency electrostimulation showed no difference between active and sham groups for pain, although cold arthralgia was significantly better in the active treatment group (Song et al., 2020). Scrambler therapy, a form of eStim, was used in one study and significantly reduced pain and improved quality of life (Coyne et al., 2013). Thermal therapies such as frozen gloves have been investigated primarily for their preventive effects. Thermoregulatory effects delivered by cryocompression were well-tolerated and were found to significantly reduce the incidence of CIPN (Bandla et al., 2020; Oneda et al., 2020; Chitkumarn et al., 2022). One study has investigated the benefit of combination therapy and implemented a 15-week integrated program of massage, passive mobilization and physical exercises with or without whole body vibration and patients reported less pain and improved function over time (Schönsteiner et al., 2017). Somatic yoga, reflexology and meditation have been tested with some influence on various measures of outcome (Kurt and Can, 2018; Galantino et al., 2019). In common with all these studies is the disparate nature in outcomes across various measures of symptoms and signs of CIPN. Although some methods and approaches show promise, more rigorously controlled studies with greater statistical power are needed. We propose that a more complete understanding of the mechanism of CIPN may assist in focusing efforts on designing non-pharmacological studies. Indeed, it may prove that combining some of the treatment methods spatially and temporally may address some of the different features contributing to the causes of CIPN.

The diversity of effectiveness of both pharmacological and non-pharmacological approaches to the prevention and management of CIPN suggests that a precision medicine approach tailored to an individual’s CIPN phenotype, physical capabilities, co-morbidities and willingness to comply with treatment may be needed, therefore accurate profiling of the individual’s CIPN would need to be developed (Dorsey et al., 2019; Mezzanotte et al., 2022).

## 10 Discussion

Our understanding of the pathomechanisms leading to CIPN have improved greatly in recent years. In particular, combination of optimized chemotherapy regimens with molecular or personalized treatment approaches could lead to development of improved treatments for CIPN with the promise to deliver improved patient outcomes. Some of the key considerations that have become apparent include the need for agent-specific, mechanisms-based treatments. These will likely include approaches targeting both aberrant neuronal function, as well as pathological processes such as inflammation that may indirectly affect sensory systems. Although adult rodent models remain invaluable in this regard, there is also a clear need for juvenile rodent models and humanized, or human, model systems that will facilitate clinical translation. One such approach may include the use of human DRGs, or stem-cell derived sensory neurons that can facilitate investigation of pathological mechanisms in more directly relevant model systems. Importantly, this review has highlighted the dearth of evidence specific to CIPN mechanisms and treatments in the pediatric population. Additional research is desperately needed, particularly in light of the clear physiological differences between children and adults that are likely to contribute to divergent mechanisms, and accordingly divergent treatment approaches, that are needed for individuals suffering from CIPN.
